# Perioperative outcomes, surgeon-rated fatigue, and scar satisfaction after dual-port versus single-port laparoscopic myomectomy: a propensity score–matched cohort study

**DOI:** 10.1186/s12905-026-04795-3

**Published:** 2026-08-18

**Authors:** Ying Liu, Yaojie Li, Yunyi Su, Xiaoyan Liao, Lijuan Xu, Aijie Xie, Yan Liu, Xiaoqin Gan

**Affiliations:** Department of Obstetrics and Gynecology, School of Medicine, Chengdu Women’s and Children’s Central Hospital, University of Electronic Science and Technology of China, 1617 Riyue Avenue, Qingyang District, Chengdu, Sichuan 611731 China

**Keywords:** Laparoscopic myomectomy, Uterine fibroids, Single-port laparoscopy, Dual-port laparoscopy, Reduced-port surgery, Propensity score matching, Surgeon fatigue, Scar satisfaction

## Abstract

**Background:**

Laparoscopic myomectomy is commonly used for women with symptomatic uterine fibroids who wish to preserve the uterus. Single-port laparoscopy reduces the number of abdominal incisions but may increase instrument crowding and ergonomic difficulty during myometrial dissection and suturing. Dual-port laparoscopy may provide a practical balance between reduced-port access and operative maneuverability. This study compared perioperative outcomes, surgeon-rated fatigue, and patient-reported scar satisfaction between dual-port and single-port laparoscopic myomectomy.

**Methods:**

This retrospective cohort study included women who underwent elective laparoscopic myomectomy for solitary uterine fibroids at a tertiary center between January 2024 and November 2025. Propensity scores were estimated using age, body mass index, fibroid diameter, fibroid location, pelvic adhesion grade, medical comorbidity, and number of previous pelvic surgeries. One-to-one nearest-neighbor matching without replacement was performed with a caliper of 0.2 standard deviations of the logit of the propensity score. Primary multivariable models in the matched cohort additionally adjusted for operating surgeon and year of surgery and used matched-pair clustered robust variance estimation. Alternative-model sensitivity analyses and regression diagnostics were also performed.

**Results:**

After propensity score matching, 190 women were included, with 95 patients in each group. Estimated blood loss was lower in the dual-port group (mean ± standard deviation, 45.53 ± 38.12 vs. 76.32 ± 69.94 mL; median [interquartile range], 30.0 [20.0–50.0] vs. 50.0 [30.0–100.0] mL). In the fully adjusted matched-cohort model, dual-port surgery remained associated with lower blood loss (β = −23.07 mL, 95% CI − 39.14 to − 7.01; *P* = 0.005), whereas the operative-time association was attenuated (β = −0.13 h, 95% CI − 0.27 to 0.00; *P* = 0.053). Surgeon-rated fatigue was available for 110 of 190 patients (57.9%) and scar satisfaction for 78 of 190 patients (41.1%); both were analyzed as exploratory complete-case outcomes. Postoperative recovery outcomes showed no consistent between-group pattern, and only one postoperative complication occurred.

**Conclusions:**

In this propensity score–matched cohort, dual-port laparoscopic myomectomy was associated with lower estimated blood loss after adjustment for operating surgeon and year of surgery. The operative-time finding was suggestive but not independently confirmed after full adjustment. Lower surgeon-rated fatigue and lower patient-reported scar-satisfaction scores were observed in exploratory complete-case analyses, but these subjective findings require confirmation using validated instruments and standardized prospective collection.

**Supplementary Information:**

The online version contains supplementary material available at https://doi.org/10.1186/s12905-026-04795-3.

## Background

Uterine fibroids are the most common benign tumors among women of reproductive age and are a frequent cause of symptoms requiring medical or surgical treatment [1–3]. For women with symptomatic fibroids who wish to preserve the uterus, laparoscopic myomectomy is widely used because it allows removal of the fibroid while maintaining the benefits of minimally invasive surgery [4].

Reduced-port laparoscopic techniques have been increasingly adopted in gynecologic surgery, partly because of their potential to reduce abdominal wall trauma and improve cosmetic outcomes [5]. However, myomectomy differs from many other reduced-port procedures because it often requires adequate traction, precise dissection, reliable hemostasis, multilayer uterine closure, and safe specimen retrieval. Therefore, reducing the number of ports does not necessarily translate into better overall surgical performance.

Single-port laparoscopy represents the most reduced form of laparoscopic access, but the use of a single umbilical incision may lead to instrument crowding, loss of triangulation, restricted range of motion, and less favorable ergonomics during myomectomy [6–8]. These limitations may be particularly relevant during myometrial suturing and uterine reconstruction. Dual-port laparoscopic myomectomy, which adds a small auxiliary port to the umbilical access, may improve instrument separation, partially restore triangulation, and facilitate traction and suturing while retaining many features of reduced-port surgery [9]. Improved instrument handling may also reduce surgeon workload and fatigue [10, 11].

Although both single-port and dual-port approaches are used in laparoscopic myomectomy, direct comparative evidence remains limited. In particular, few studies have evaluated not only perioperative outcomes but also surgeon-rated fatigue and patient-reported scar satisfaction, which are relevant to both surgical performance and patient-centered care. To address this gap, we compared dual-port and single-port laparoscopic myomectomy in women with solitary uterine fibroids using propensity score matching, multivariable regression, and sensitivity analyses.

## Methods

### Study design and participants

**Fig. 1:**
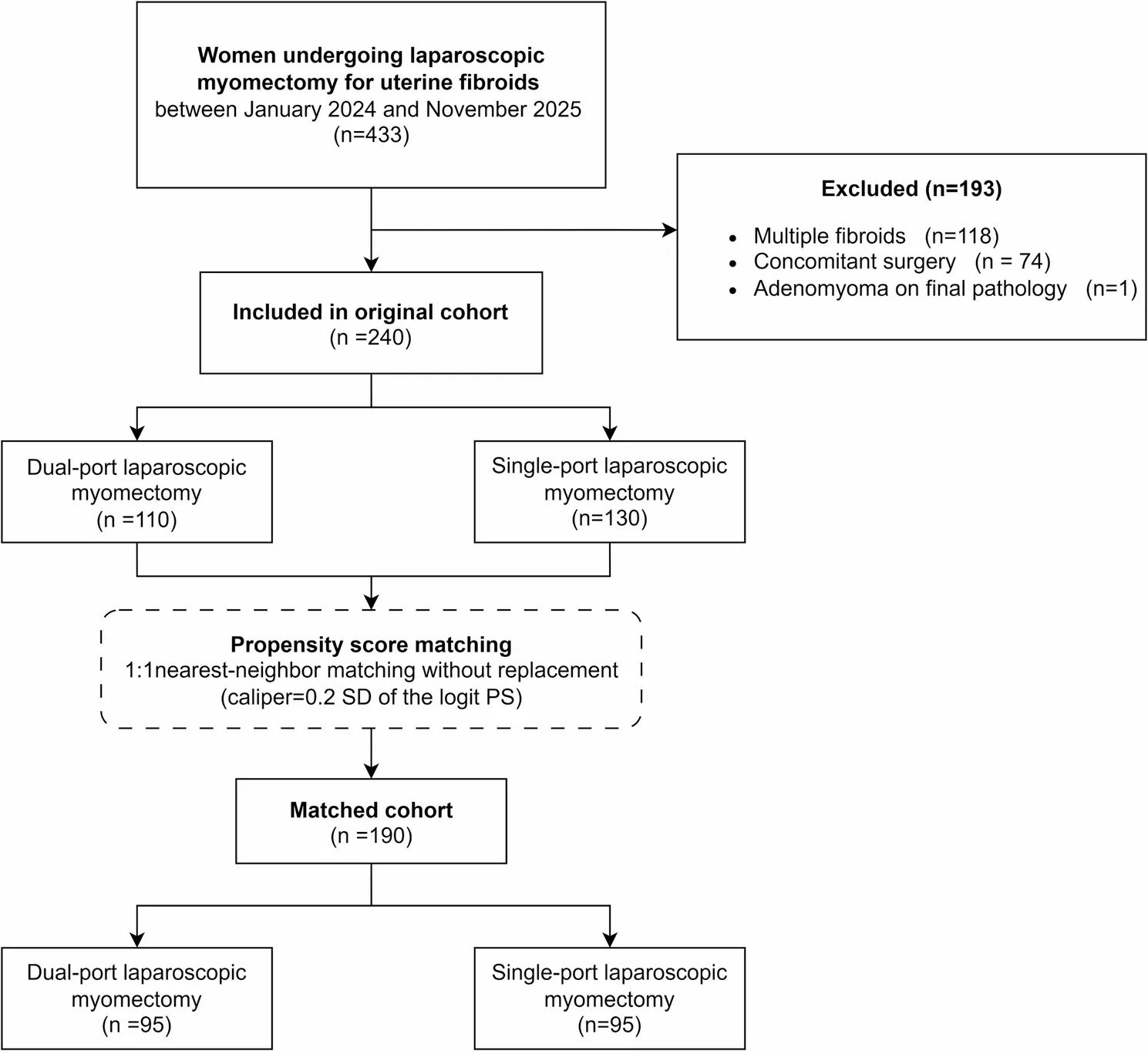
Flowchart of patient selection and propensity score matching. A total of 433 women were screened. Of the 193 excluded women, 118 had multiple fibroids, 74 underwent concomitant procedures, and 1 patient who underwent single-port surgery was excluded because final postoperative pathology demonstrated adenomyoma. The original cohort included 240 women (130 single-port and 110 dual-port). Primary propensity score matching retained 95 patients in each group

This retrospective cohort study was conducted at Chengdu Women’s and Children’s Central Hospital, a tertiary center for minimally invasive gynecologic surgery, between January 2024 and November 2025. The study protocol was approved by the Ethics Committee of Chengdu Women’s and Children’s Central Hospital (No. 2022207). Women aged 18 years or older who underwent elective laparoscopic myomectomy during the study period were screened. Patients were eligible if they had a presumed solitary uterine fibroid based on preoperative ultrasonography. Final clinical and pathological findings were reviewed to confirm eligibility. Patients were excluded for multiple fibroids requiring simultaneous removal, concomitant surgical procedures, adenomyosis or adenomyoma, malignancy, or incomplete essential records. Of 433 screened women, 193 were excluded: 118 because of multiple fibroids, 74 because of concomitant procedures, and 1 because final postoperative pathology demonstrated adenomyoma following single-port surgery. No patient was excluded because of malignancy or incomplete essential records. The original cohort therefore included 240 patients (110 dual-port and 130 single-port), and the primary matched cohort included 190 patients (95 per group) (Fig. 1).

### Surgical procedures

Three attending gynecologic laparoscopists performed all procedures, and each surgeon used both techniques during the study period. All surgeons had more than 10 years of experience in minimally invasive gynecologic surgery. The surgical approach was selected preoperatively according to fibroid characteristics, patient factors, cosmetic preference, and anticipated technical feasibility. Operating surgeon and year of surgery were included in the adjusted analyses, and a separate propensity-score sensitivity analysis also incorporated these variables.

In the single-port group, a 2–2.5-cm umbilical incision and multichannel access device were used. In the dual-port group, the same umbilical access was supplemented by a 5-mm lower-abdominal port for traction, exposure, suturing assistance, electrocautery, and suction or irrigation.

Before uterine incision, 6 units of pituitrin diluted in 20 mL of normal saline were injected into the surrounding myometrium after negative aspiration, with the total dose not exceeding 6 units. Uterine tourniquets and temporary vascular occlusion were not routinely used. The uterine incision was made with monopolar electrosurgery, and bipolar forceps were used selectively for focal hemostasis.

The myometrial defect was closed in two or three layers using 0-gauge absorbable barbed sutures, according to defect depth and extent. When the endometrial cavity was entered, it was closed separately before myometrial reconstruction. Fibroids were placed in a retrieval bag, manually fragmented within the bag, and removed through the umbilical incision. No uncontained morcellation was performed.

The same hemostatic, energy-device, suturing, and specimen-extraction principles were applied in both groups. No procedure required incision enlargement, additional ports, conversion to conventional multiport laparoscopy or laparotomy, or modification of the planned port configuration. No included patient received preoperative gonadotropin-releasing hormone agonists, gonadotropin-releasing hormone antagonists, or other fibroid-directed medical therapy intended to reduce fibroid size. Detailed anatomical variables such as FIGO subtype, intramural depth, cavity relationship, and procedure-specific reconstruction requirements were not consistently available and could not be included in the adjusted analyses. Residual confounding related to unmeasured operative complexity therefore remains possible.

### Data collection and variables

Clinical and perioperative data were extracted from electronic medical records. Baseline variables included age, body mass index, medical comorbidity, number of previous pelvic surgeries, fibroid diameter, fibroid location, pelvic adhesion grade, operating surgeon, and year of surgery. Fibroid diameter was defined as the largest dimension reported on the most recent preoperative pelvic ultrasonography; when magnetic resonance imaging was also available, the ultrasound measurement was retained to ensure a consistent source. Fibroid location was categorized as posterior wall or non-posterior wall. Medical comorbidity was coded as a binary composite variable indicating at least one documented pre-existing chronic condition, including but not limited to hypothyroidism, hyperthyroidism, hypertension, diabetes mellitus, cardiovascular disease, or respiratory disease. Pelvic adhesions were graded intraoperatively by the operating surgeon using a pragmatic four-level institutional classification (grade 0, none; grade 1, mild; grade 2, moderate; grade 3, severe) and were analyzed as an ordinal variable. This grading system was not formally validated, and no interobserver calibration or reliability assessment was performed.

Perioperative outcomes included operative time, estimated blood loss, postoperative pain score, analgesic use within 24 h, postoperative recovery indicators, length of hospital stay, and postoperative complications. Operative time was defined from skin incision to skin closure. Estimated blood loss was calculated as total suction-canister volume minus documented irrigation-fluid volume. Swabs, gauze, and drapes were not routinely weighed, and blood outside the suction system was not quantified. The same institutional estimation method was used throughout the study period and across surgeons, although operating-room personnel varied according to routine scheduling. Postoperative recovery indicators included time to first oral intake, ambulation, spontaneous voiding, and flatus. Indwelling urinary catheters were not routinely used during or after surgery; patients emptied their bladder before entering the operating room. Postoperative complications were defined as adverse events occurring within 30 days after surgery.

Postoperative pain was assessed 2 h after surgery using a visual analogue scale, and analgesic use within 24 h was recorded as a binary variable. Surgeon-rated fatigue was recorded immediately after the procedure using a non-validated single-item five-point overall subjective rating, with higher scores indicating greater fatigue. The item did not distinguish physical fatigue, mental workload, technical difficulty, or emotional stress; baseline fatigue, time of day, preceding workload, and earlier procedures on the same day were not systematically recorded. Patient-reported scar satisfaction was recorded during postoperative follow-up using a non-validated single-item 10-point numerical rating, with lower scores indicating greater satisfaction. The assessment timing was not consistently standardized. These assessments were optional components of routine care rather than mandatory research measures, were not retrospectively reconstructed, and were analyzed as exploratory complete-case outcomes.

### Propensity score matching

Propensity scores were estimated using logistic regression with surgical approach as the dependent variable and age, body mass index, fibroid diameter, fibroid location, pelvic adhesion grade, medical comorbidity, and number of previous pelvic surgeries as covariates. One-to-one nearest-neighbor matching without replacement was performed using MatchIt, with the average treatment effect in the treated estimand and a caliper of 0.20 standard deviations of the logit of the propensity score. Of the original 130 single-port and 110 dual-port patients, 95 patients from each group were retained, corresponding to 73.1% of single-port and 86.4% of dual-port patients; 35 single-port and 15 dual-port patients were unmatched and excluded. Before matching, propensity scores ranged from 0.107 to 0.918 in the single-port group and from 0.147 to 0.979 in the dual-port group, with sufficient overlap and no clear positivity violation. No random sampling was used; the matched sample was determined by the specified nearest-neighbor algorithm and the order of observations in the analytical dataset, which was retained for reproducibility.

Covariate balance before and after matching was evaluated using absolute standardized mean differences, with values below 0.10 considered indicative of acceptable balance. Baseline characteristics of the primary matched cohort are presented in Table 1, and complete baseline characteristics before and after primary matching are provided in Supplementary Table S1. A separate sensitivity propensity-score analysis incorporating operating surgeon and year of surgery yielded 72 matched pairs; its balance diagnostics and multivariable outcome analyses are reported in Supplementary Tables S2 and S3, respectively. No missing fatigue or scar-satisfaction scores were imputed. Covariate balance and propensity-score distributions are shown in Supplementary Figures S1 and S3, respectively.

### Statistical analysis

All analyses were performed using R software, version 4.6.0 (R Foundation for Statistical Computing, Vienna, Austria), including MatchIt for propensity-score matching and clubSandwich for matched-cluster robust variance estimation. Because this retrospective cohort included all eligible patients during the study period, no formal a priori sample-size calculation was performed.

Continuous variables are presented as mean ± standard deviation, with median and interquartile range additionally reported for clearly skewed estimated blood loss. Categorical variables are presented as frequencies and percentages. Baseline balance was summarized using standardized mean differences rather than hypothesis tests. Outcome comparisons were exploratory, and all reported P values are nominal; no multiplicity adjustment was applied. Effect estimates and 95% confidence intervals were prioritized over isolated P values.

Post-matching multivariable regression was performed to further adjust for measured confounding, particularly operating surgeon and year of surgery, which were not included in the primary propensity-score model. Models were fitted separately for estimated blood loss, operative time, surgeon-rated fatigue, and patient-reported scar satisfaction, adjusting for surgical approach, baseline covariates, operating surgeon, and year of surgery. Robust standard errors were clustered by matched pair using the MatchIt subclass identifier with small-sample correction. Model-specific sample sizes were 189, 190, 110, and 78, respectively.

Regression diagnostics included residual Q–Q plots, Shapiro–Wilk tests, Breusch–Pagan tests, Cook’s distance, and variance inflation factors or adjusted generalized variance inflation factors. Sensitivity analyses comprised log-transformed linear regression for estimated blood loss and ordinal logistic regression for the five-point fatigue and 10-point scar-satisfaction scores, with matched-pair clustered robust standard errors. Because score availability showed substantial temporal variation and determinants of completion were incompletely observed, missing outcome scores were not imputed and inverse-probability weighting was not used.

All tests were two-sided. P values below 0.001 are reported as *P* < 0.001; other P values are reported exactly where available.

## Results

### Patient characteristics

A total of 433 women were screened. Of the 193 excluded women, 118 had multiple fibroids, 74 underwent concomitant procedures, and 1 single-port patient was excluded after final pathology demonstrated adenomyoma. The original cohort comprised 130 single-port and 110 dual-port procedures. Primary propensity-score matching retained 95 patients in each group. Baseline characteristics of the primary matched cohort are shown in Table 1, and complete baseline characteristics before and after matching are provided in Supplementary Table S1. Fibroid diameter showed substantial imbalance before matching (SMD = 0.441), whereas all reported baseline covariates had absolute SMDs below 0.10 after matching (Table 1 and Supplementary Figure S1).

**Table 1:** Baseline characteristics of the propensity-score–matched population

Variable	Single-port group (*n* = 95)	Dual-port group (*n* = 95)	SMD
Age, years	37.79 ± 7.81	38.01 ± 7.38	0.029
BMI, kg/m²	22.90 ± 3.50	22.58 ± 3.33	0.095
Fibroid diameter, cm	6.60 ± 1.40	6.64 ± 1.39	0.030
Fibroid location (posterior)	39 (41.1%)	40 (42.1%)	0.021
Pelvic adhesion grade	0.57 ± 0.79	0.56 ± 0.77	0.013
Medical comorbidity, n (%)	5 (5.3%)	5 (5.3%)	0.000
Number of previous pelvic surgeries	0.58 ± 0.87	0.65 ± 0.75	0.090

Data are presented as mean ± standard deviation or n (%), as appropriate

*BMI *body mass index, *SMD *standardized mean difference

### Perioperative outcomes

**Fig. 2:**
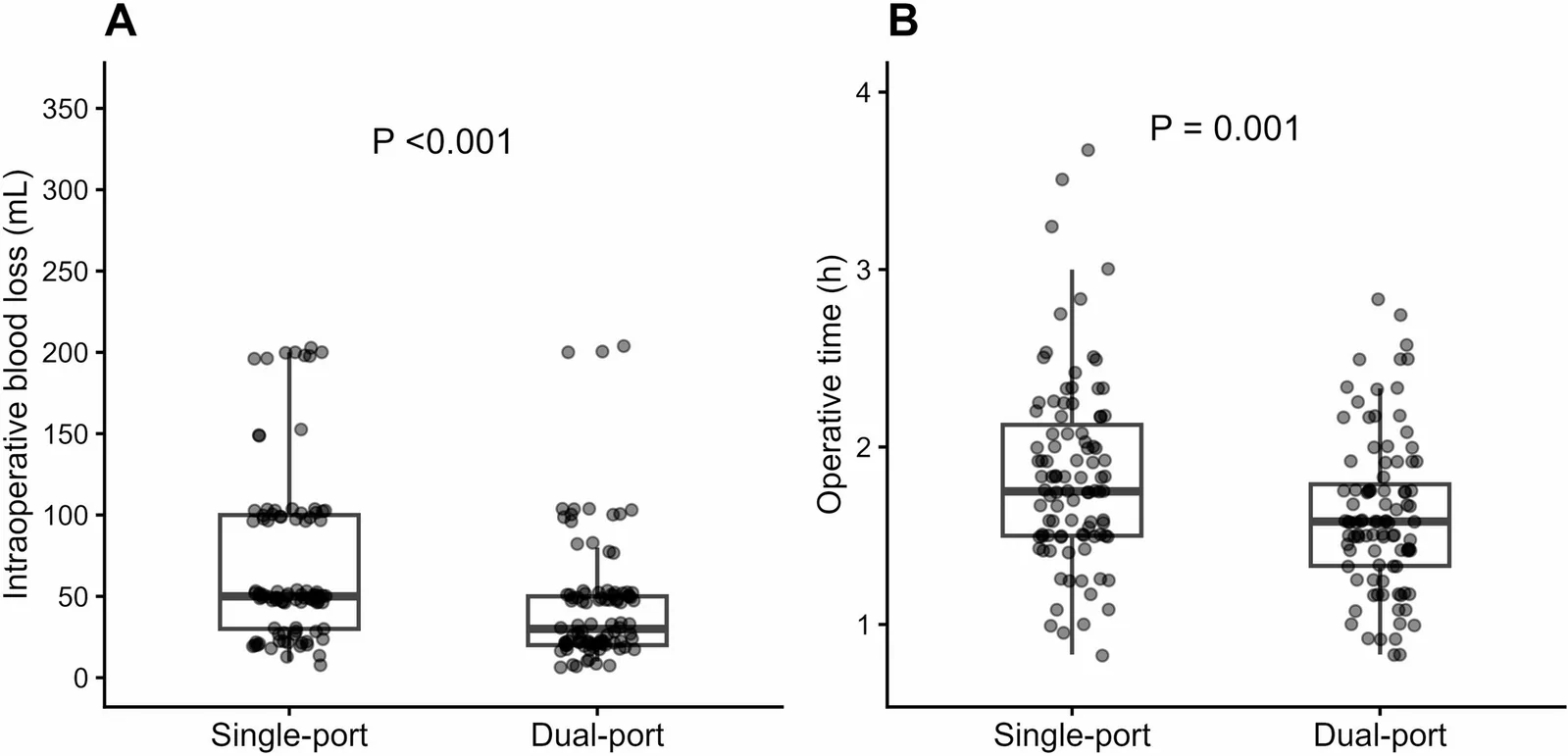
Intraoperative outcomes in the propensity-score–matched cohort. **A** Estimated intraoperative blood loss. **B** Operative time. Data are presented as box-and-whisker plots with individual observations. Boxes represent the interquartile range (IQR), center lines indicate the median, and whiskers extend to 1.5 × IQR

Perioperative outcomes are summarized in Table 2. Estimated blood loss was lower in the dual-port group (mean ± standard deviation, 45.53 ± 38.12 vs. 76.32 ± 69.94 mL; median [interquartile range], 30.0 [20.0–50.0] vs. 50.0 [30.0–100.0] mL). Operative time was also shorter in the unadjusted matched-cohort comparison (1.62 ± 0.44 vs. 1.85 ± 0.52 h; *P* = 0.001). The distributions of estimated blood loss and operative time are shown in Fig. 2. Postoperative recovery outcomes showed no consistent between-group pattern; the isolated nominal difference in time to first voiding (*P* = 0.041) was not interpreted as definitive because multiple exploratory comparisons were performed. Fatigue-score availability differed markedly by year of surgery (92.2% in 2024 vs. 27.0% in 2025; *P* < 0.001) but not by surgical approach or operating surgeon (Supplementary Table S6). Scar-satisfaction availability showed no clear differences according to surgical approach, operating surgeon, year of surgery, baseline characteristics, or perioperative outcomes (Supplementary Table S7). One postoperative pelvic infection occurred in the dual-port group on postoperative day 3 and was managed with readmission and intravenous antibiotics; it was classified as Clavien–Dindo grade II.

**Table 2:** Perioperative outcomes in the propensity-score-matched cohort

Variable	Single-port group (*n* = 95)	Dual-port group (*n* = 95)	*P* value
Intraoperative blood loss (mL)	76.32 ± 69.94	45.53 ± 38.12	< 0.001
Median (IQR)	50.0 (30.0–100.0)	30.0 (20.0–50.0)	
Operative time (h)	1.85 ± 0.52	1.62 ± 0.44	0.001
Time to first oral intake (h)	2.24 ± 1.62	2.27 ± 0.92	0.856
Time to first ambulation (h)	2.79 ± 0.93	2.99 ± 0.97	0.143
Time to first voiding (h)	3.01 ± 0.99	3.33 ± 1.09	0.041
Time to first flatus (h)	11.87 ± 4.50	11.45 ± 5.30	0.597
Length of hospital stay (days)	1.04 ± 0.25	1.02 ± 0.14	0.477
Pain score (VAS)	1.78 ± 0.91	1.69 ± 0.67	0.467
Surgeon-rated fatigue scoreᵃ	2.84 ± 0.77 (*n* = 58)	1.73 ± 0.66 (*n* = 52)	< 0.001
Patient-reported scar satisfaction scoreᵇ	6.72 ± 3.54 (*n* = 36)	4.12 ± 3.70 (*n* = 42)	0.002
Analgesic use, n (%)	0 (0.0%)	2 (2.1%)	0.497
Complications, n (%)	0 (0.0%)	1 (1.1%)	1.000

Estimated blood loss is presented as both mean ± standard deviation and median (interquartile range) because of its right-skewed distribution. Mean ± standard deviation is retained because the primary regression analyses estimate mean differences. Blood-loss data were available for all 95 patients in the single-port group and 94 of 95 patients in the dual-port group. The reported P value for estimated blood loss corresponds to the primary mean-based comparison; median and interquartile range are provided descriptively because of the right-skewed distribution

*VAS* visual analogue scale

ᵃ Surgeon-rated fatigue score was analyzed based on available cases; lower scores indicate less surgeon fatigue

ᵇ Patient-reported scar satisfaction score was analyzed based on available cases; lower scores indicate greater scar satisfaction

### Multivariable regression analyses

**Fig. 3:**
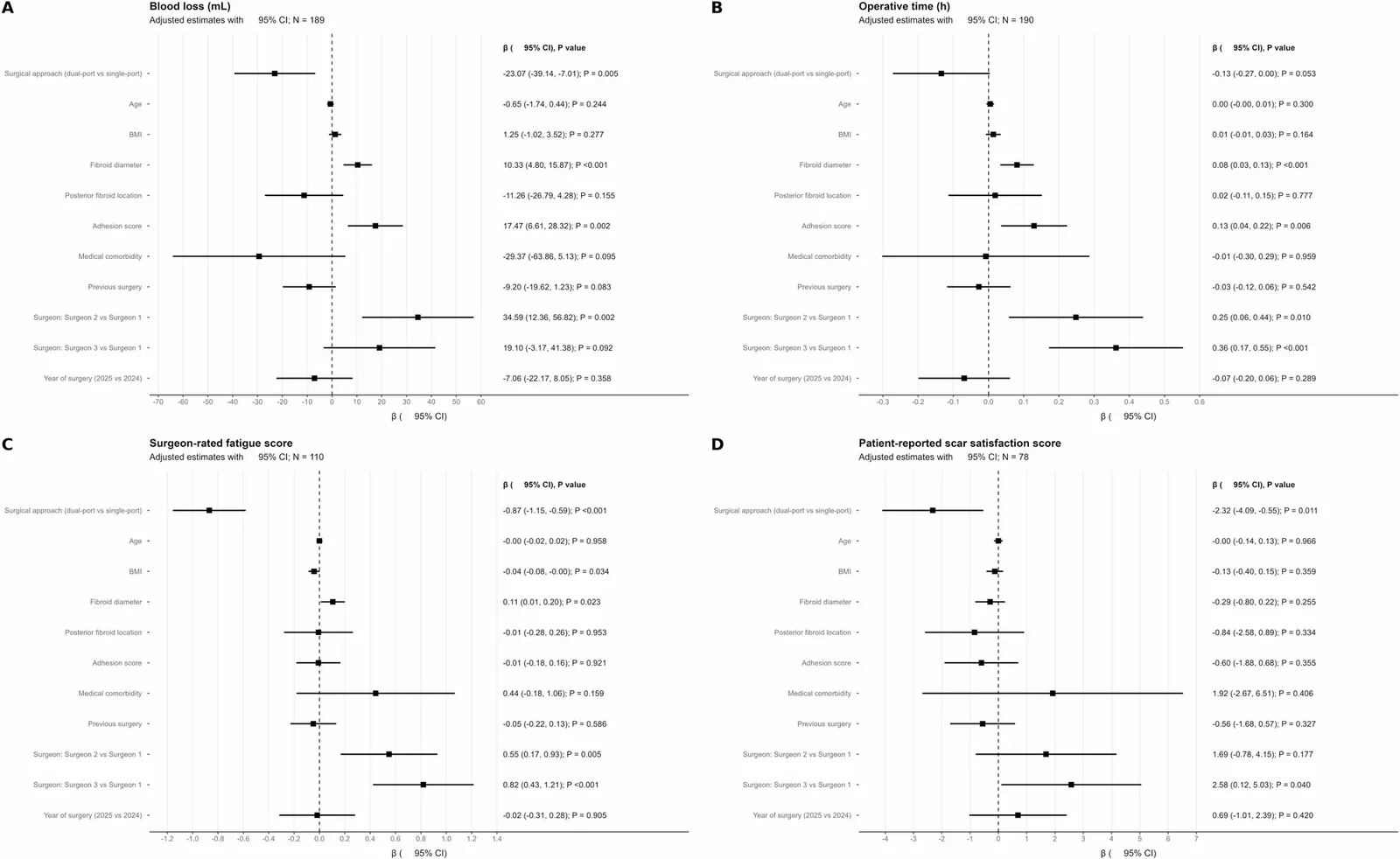
Adjusted associations of surgical approach and covariates with perioperative outcomes in the propensity-score–matched cohort. Forest plots show adjusted β coefficients and 95% confidence intervals for **A **estimated intraoperative blood loss, **B** operative time, **C **surgeon-rated fatigue score, and **D** patient-reported scar satisfaction score. The vertical dashed line indicates no association (β = 0). Each model included surgical approach, age, body mass index, fibroid diameter, posterior fibroid location, pelvic adhesion grade, medical comorbidity, previous pelvic surgery, operating surgeon, and year of surgery. Single-port laparoscopy, Surgeon 1, and year 2024 were used as the reference categories, as applicable. Lower fatigue and scar-satisfaction scores indicate less surgeon fatigue and greater scar satisfaction, respectively

Fully adjusted multivariable results are presented in Table 3; Fig. 3. After adjustment for baseline covariates, operating surgeon, and year of surgery, dual-port surgery remained associated with lower estimated blood loss (β = −23.07 mL, 95% CI − 39.14 to − 7.01; *P* = 0.005). The operative-time estimate was attenuated and its confidence interval included the null (β = −0.13 h, 95% CI − 0.27 to 0.00; *P* = 0.053). In the exploratory complete-case models, dual-port surgery was associated with lower surgeon-rated fatigue (β = −0.87, 95% CI − 1.15 to − 0.59; *P* < 0.001) and lower scar-satisfaction scores (β = −2.32, 95% CI − 4.09 to − 0.55; *P* = 0.011). Operating surgeon was independently associated with several outcomes, underscoring the importance of surgeon adjustment.

**Table 3:** Multivariable regression analyses in the propensity-score-matched cohort

Variable	Blood loss (mL), β (95% CI), *N* = 189	*P* value	Operative time (h), β (95% CI), *N* = 190	*P* value	Surgeon-rated fatigue score, β (95% CI), *N* = 110	*P* value	Patient-reported scar satisfaction score, β (95% CI), *N* = 78	*P* value
Surgical approach (dual-port vs. single-port)	-23.07 (-39.14, -7.01)	0.005	-0.13 (-0.27, 0.00)	0.053	-0.87 (-1.15, -0.59)	< 0.001	-2.32 (-4.09, -0.55)	0.011
Age	-0.65 (-1.74, 0.44)	0.244	0.00 (-0.00, 0.01)	0.300	-0.00 (-0.02, 0.02)	0.958	-0.00 (-0.14, 0.13)	0.966
BMI	1.25 (-1.02, 3.52)	0.277	0.01 (-0.01, 0.03)	0.164	-0.04 (-0.08, -0.00)	0.034	-0.13 (-0.40, 0.15)	0.359
Fibroid diameter	10.33 (4.80, 15.87)	< 0.001	0.08 (0.03, 0.13)	< 0.001	0.11 (0.01, 0.20)	0.023	-0.29 (-0.80, 0.22)	0.255
Posterior fibroid location	-11.26 (-26.79, 4.28)	0.155	0.02 (-0.11, 0.15)	0.777	-0.01 (-0.28, 0.26)	0.953	-0.84 (-2.58, 0.89)	0.334
Pelvic adhesion grade	17.47 (6.61, 28.32)	0.002	0.13 (0.04, 0.22)	0.006	-0.01 (-0.18, 0.16)	0.921	-0.60 (-1.88, 0.68)	0.355
Medical comorbidity	-29.37 (-63.86, 5.13)	0.095	-0.01 (-0.30, 0.29)	0.959	0.44 (-0.18, 1.06)	0.159	1.92 (-2.67, 6.51)	0.406
Previous pelvic surgery	-9.20 (-19.62, 1.23)	0.083	-0.03 (-0.12, 0.06)	0.542	-0.05 (-0.22, 0.13)	0.586	-0.56 (-1.68, 0.57)	0.327
Surgeon: Surgeon 2 vs. Surgeon 1	34.59 (12.36, 56.82)	0.002	0.25 (0.06, 0.44)	0.010	0.55 (0.17, 0.93)	0.005	1.69 (-0.78, 4.15)	0.177
Surgeon: Surgeon 3 vs. Surgeon 1	19.10 (-3.17, 41.38)	0.092	0.36 (0.17, 0.55)	< 0.001	0.82 (0.43, 1.21)	< 0.001	2.58 (0.12, 5.03)	0.040
Year of surgery (2025 vs. 2024)	-7.06 (-22.17, 8.05)	0.358	-0.07 (-0.20, 0.06)	0.289	-0.02 (-0.31, 0.28)	0.905	0.69 (-1.01, 2.39)	0.420

The single-port group, Surgeon 1, and the year 2024 were used as the reference categories for surgical approach, surgeon, and year of surgery, respectively. Regression models were adjusted for surgical approach, age, BMI, fibroid diameter, posterior fibroid location, pelvic adhesion grade, medical comorbidity, previous pelvic surgery, operating surgeon, and year of surgery. Model-specific sample sizes are reported in the column headings

*BMI *body mass index, *CI* confidence interval

### Sensitivity analyses

Alternative-model sensitivity analyses are summarized in Supplementary Table S4 and Supplementary Figure S2. The sensitivity propensity-score analysis incorporating operating surgeon and year of surgery is reported in Supplementary Tables S2 and S3. In the log-transformed blood-loss model, dual-port surgery was associated with a lower geometric mean (GMR = 0.756, 95% CI 0.614–0.930; *P* = 0.009). Ordinal logistic regression produced consistent directional findings for fatigue (OR = 0.052, 95% CI 0.012–0.227; *P* < 0.001) and scar satisfaction (OR = 0.336, 95% CI 0.129–0.876; *P* = 0.026). In the 72-pair sensitivity-matched cohort, lower blood loss remained significant (β = −16.26 mL, 95% CI − 31.60 to − 0.92; *P* = 0.038), whereas operative time was not (β = −0.09 h, 95% CI − 0.23 to 0.04; *P* = 0.181). Diagnostic assessment indicated non-normality and potential heteroscedasticity for the blood-loss and fatigue models, but no observation had Cook’s distance above 1 and multicollinearity was acceptable (Supplementary Table S5).

## Discussion

In this propensity score–matched cohort, dual-port laparoscopic myomectomy was associated with lower estimated blood loss after adjustment for operating surgeon, year of surgery, measured patient characteristics, and the available indicators of operative complexity. In contrast, the association with operative time was attenuated after full adjustment and did not reach conventional statistical significance. The blood-loss result remained directionally consistent across the sensitivity analyses (Supplementary Tables S2–S4). However, the clinical importance of this finding remains uncertain because the absolute difference was modest, no patient required blood transfusion, blood loss was estimated rather than directly measured, and postoperative recovery was not consistently improved.

These findings should therefore be interpreted as adjusted observational associations rather than evidence that port configuration alone determines perioperative outcomes.

Reduced-port laparoscopy should not be considered a uniform surgical technique. A single umbilical access can increase instrument crowding and restrict triangulation, whereas an auxiliary port may improve instrument separation, traction, and suturing angles [9–10, 12]. These technical differences provide a plausible explanation for the lower estimated blood loss observed with dual-port surgery, but they do not establish causality in this non-randomized study.

The unadjusted matched-cohort comparison suggested shorter operative time with dual-port surgery. However, operating surgeon was strongly associated with operative time, and the fully adjusted estimate was attenuated and imprecise. This suggests that the observed difference may reflect surgeon-related factors, including individual experience and procedural preference, at least as much as port configuration itself. The operative-time finding should therefore be regarded as suggestive rather than independently confirmed.

Published comparisons of single-port, reduced-port, and conventional laparoscopy have yielded heterogeneous results for operative time, blood loss, complications, and patient-reported outcomes [13–21]. The present study adds evidence regarding a two-port approach but should not be interpreted as establishing its overall superiority. Selection of surgical approach should account for patient preference, fibroid characteristics, anticipated reconstruction requirements, and surgeon experience.

Larger fibroid diameter and higher pelvic adhesion grade were associated with greater blood loss and longer operative time. However, the available variables did not capture FIGO classification, exact intramural depth, relationship to the endometrial cavity, fibroid or uterine volume, vascularity, pseudocapsule characteristics, or detailed reconstruction requirements. In addition, the institutional adhesion grading system was not formally validated and may have been subject to interobserver variation. Residual confounding from incompletely measured operative complexity therefore remains possible.

Lower surgeon-rated fatigue was observed in the dual-port group, but this finding was based on a non-validated single-item score available for only 110 patients. Fatigue-score availability differed significantly by year of surgery but not by surgical approach, operating surgeon, or baseline characteristics (Supplementary Table S6). The marked difference in fatigue-score availability between 2024 and 2025 (92.2% vs. 27.0%) suggests that the missing data were unlikely to be missing completely at random and may reflect temporal changes in documentation practices. Surgeon fatigue is influenced by multiple ergonomic and procedural factors beyond surgical approach, including baseline workload, time of day, preceding procedures, and individual response tendencies, many of which were not systematically recorded in the present study [10–11, 22]. This result should therefore be interpreted as exploratory rather than as evidence that dual-port surgery is ergonomically superior.

Lower patient-reported scar-satisfaction scores, indicating greater satisfaction, were observed in the dual-port group in an exploratory complete-case analysis of 78 patients. Supplementary Table S7 showed that scar-satisfaction score availability was not significantly associated with surgical approach, operating surgeon, year of surgery, baseline characteristics, or perioperative outcomes. The non-validated single-item rating did not separately assess scar appearance, symptoms, texture, visibility, or psychosocial impact [23–24], and the timing of follow-up was not consistently standardized. Moreover, preoperative cosmetic preference may have influenced both selection of the surgical approach and subsequent scar evaluation. The model included many covariates relative to the available sample and may therefore have been imprecise or overfitted. Consequently, this result does not establish a clinically meaningful cosmetic advantage.

Postoperative recovery outcomes showed no consistent pattern favoring either approach. The nominal difference in time to first voiding may represent a chance finding in the context of multiple comparisons. Only one postoperative complication occurred; therefore, although complications were uncommon in this cohort, the study was not adequately powered to compare uncommon adverse events or establish equivalent safety. Regression diagnostics did not identify influential observations, substantial multicollinearity, or other findings likely to materially alter interpretation of the primary linear regression models, although potential heteroskedasticity was observed for the blood-loss and fatigue models (Supplementary Table S5).

Several limitations should be acknowledged. First, this retrospective single-center study remains vulnerable to confounding by indication, surgeon preference, temporal changes in practice, and patient preference. Although operating surgeon and year of surgery were included in the adjusted analyses, the distribution of surgical approaches was uneven before matching, and calendar year is only an imperfect surrogate for individual learning experience. Second, estimated blood loss was based on suction-canister volume minus irrigation fluid without weighing swabs or quantifying blood outside the suction system, and hemoglobin change was not sufficiently complete for objective corroboration. Third, detailed anatomical and reconstruction-related variables, including FIGO subtype, intramural depth, cavity relationship, and procedure-specific reconstruction requirements, were not consistently available, leaving residual confounding from operative complexity. Fourth, surgeon-rated fatigue and scar satisfaction were based on non-validated single-item measures with substantial, potentially non-random missingness. Fifth, restriction to women with solitary fibroids limits generalizability to patients with multiple fibroids or more complex uterine reconstruction. Finally, the low number of complications limited safety comparisons, while the single-center setting with highly experienced surgeons limited generalizability. Future prospective multicenter studies including patients with multiple fibroids and greater operative complexity should assess validated patient- and surgeon-reported outcomes, as well as fertility, pregnancy, and other long-term reproductive outcomes [25].

In conclusion, among women with solitary uterine fibroids, dual-port laparoscopic myomectomy was associated with lower estimated blood loss in the fully adjusted propensity score–matched cohort. The operative-time result was suggestive but not independently confirmed after adjustment for operating surgeon and year of surgery. Lower surgeon-rated fatigue and lower scar-satisfaction scores were observed only in exploratory complete-case analyses and require confirmation using validated instruments, standardized data collection, and prospective multicenter designs.

## Supplementary Information

Supplementary Material 1.

## Data Availability

The data that support the findings of this study are available on request from the corresponding author. The data are not publicly available due to privacy restrictions.
