# Supplementary material for: Perioperative outcomes, surgeon-rated fatigue, and scar satisfaction after dual-port versus single-port laparoscopic myomectomy: a propensity score–matched cohort study
Source: BMC Womens Health. 2026 Aug 18;26:611. doi: 10.1186/s12905-026-04795-3 (PMC13761090; doi:10.1186/s12905-026-04795-3)
Supplement: Supplementary file 1 [file 12905_2026_4795_MOESM1_ESM.docx]

**Supplementary Tables**

**Supplementary Table S1. Baseline characteristics before and after primary propensity score matching**

|  | **Before matching** | | | **After matching** | | |
| --- | --- | --- | --- | --- | --- | --- |
| **Characteristic** | **single-port N = 130** | **dual-port N = 110** | **SMD** | **single-port N = 95** | **dual-port N = 95** | **SMD** |
| Age, years | 38.10 ± 8.23 | 38.71 ± 7.37 | 0.083 | 37.79 ± 7.81 | 38.01 ± 7.38 | 0.029 |
| Body mass index, kg/m² | 22.80 ± 3.39 | 22.49 ± 3.28 | 0.094 | 22.90 ± 3.50 | 22.58 ± 3.33 | 0.095 |
| Fibroid diameter, cm | 6.30 ± 1.39 | 7.03 ± 1.64 | 0.441 | 6.60 ± 1.40 | 6.64 ± 1.39 | 0.030 |
| Fibroid location, n (%) |  |  |  |  |  |  |
| Non-posterior wall | 80 (61.5%) | 63 (57.3%) |  | 56 (58.9%) | 55 (57.9%) |  |
| Posterior wall | 50 (38.5%) | 47 (42.7%) | 0.086 | 39 (41.1%) | 40 (42.1%) | 0.021 |
| Pelvic adhesion grade | 0.55 ± 0.82 | 0.60 ± 0.81 | 0.066 | 0.57 ± 0.79 | 0.56 ± 0.77 | 0.013 |
| Medical comorbidity, n (%) |  |  |  |  |  |  |
| No | 119 (91.5%) | 103 (93.6%) |  | 90 (94.7%) | 90 (94.7%) |  |
| Yes | 11 (8.5%) | 7 (6.4%) | 0.086 | 5 (5.3%) | 5 (5.3%) | 0.000 |
| Number of previous pelvic surgeries | 0.62 ± 0.87 | 0.63 ± 0.74 | 0.006 | 0.58 ± 0.87 | 0.65 ± 0.75 | 0.090 |

Data are presented as mean ± standard deviation or n (%). SMD, standardized mean difference. Absolute SMDs were calculated using the treated-group standard deviation as the denominator. For multilevel categorical variables, SMDs are reported for each indicator level; for binary variables, the SMD is displayed for the non-reference level. An absolute SMD < 0.10 was considered indicative of acceptable covariate balance.

**Supplementary Table S2. Baseline characteristics before and after sensitivity propensity score matching incorporating operating surgeon and year of surgery**

|  | **Before matching** | | | **After matching** | | |
| --- | --- | --- | --- | --- | --- | --- |
| **Characteristic** | **Single-port N = 130** | **Dual-port N = 110** | **SMD** | **Single-port N = 72** | **Dual-port N = 72** | **SMD** |
| Age, years | 38.10 ± 8.23 | 38.71 ± 7.37 | 0.083 | 39.22 ± 8.13 | 38.78 ± 7.31 | 0.060 |
| Body mass index, kg/m² | 22.80 ± 3.39 | 22.49 ± 3.28 | 0.094 | 22.73 ± 3.42 | 22.50 ± 3.01 | 0.069 |
| Fibroid diameter, cm | 6.30 ± 1.39 | 7.03 ± 1.64 | 0.441 | 6.62 ± 1.43 | 6.78 ± 1.48 | 0.096 |
| **Fibroid location, n (%)** |  |  |  |  |  |  |
| Non-posterior wall | 80 (61.5%) | 63 (57.3%) |  | 44 (61.1%) | 44 (61.1%) |  |
| Posterior wall | 50 (38.5%) | 47 (42.7%) | 0.086 | 28 (38.9%) | 28 (38.9%) | 0.000 |
| **Pelvic adhesion grade, n (%)** |  |  |  |  |  |  |
| Grade 0 | 80 (61.5%) | 63 (57.3%) | 0.086 | 44 (61.1%) | 41 (56.9%) | 0.084 |
| Grade 1 | 35 (26.9%) | 32 (29.1%) | 0.048 | 23 (31.9%) | 23 (31.9%) | 0.000 |
| Grade 2 | 9 (6.9%) | 11 (10.0%) | 0.103 | 5 (6.9%) | 7 (9.7%) | 0.093 |
| Grade 3 | 6 (4.6%) | 4 (3.6%) | 0.052 | 0 (0.0%) | 1 (1.4%) | 0.074 |
| **Medical comorbidity, n (%)** |  |  |  |  |  |  |
| No | 119 (91.5%) | 103 (93.6%) |  | 68 (94.4%) | 68 (94.4%) |  |
| Yes | 11 (8.5%) | 7 (6.4%) | 0.086 | 4 (5.6%) | 4 (5.6%) | 0.000 |
| Previous pelvic surgeries, n | 0.62 ± 0.87 | 0.63 ± 0.74 | 0.006 | 0.61 ± 0.85 | 0.62 ± 0.72 | 0.019 |
| **Surgeon, n (%)** |  |  |  |  |  |  |
| Surgeon 1 | 7 (5.4%) | 40 (36.4%) | 0.644 | 7 (9.7%) | 8 (11.1%) | 0.029 |
| Surgeon 2 | 52 (40.0%) | 37 (33.6%) | 0.135 | 31 (43.1%) | 32 (44.4%) | 0.029 |
| Surgeon 3 | 71 (54.6%) | 33 (30.0%) | 0.537 | 34 (47.2%) | 32 (44.4%) | 0.061 |
| **Year of surgery, n (%)** |  |  |  |  |  |  |
| 2024 | 67 (51.5%) | 49 (44.5%) |  | 34 (47.2%) | 34 (47.2%) |  |
| 2025 | 63 (48.5%) | 61 (55.5%) | 0.141 | 38 (52.8%) | 38 (52.8%) | 0.000 |

Data are presented as mean ± standard deviation or n (%). SMD, standardized mean difference. Absolute SMDs were calculated using the treated-group standard deviation as the denominator. For multilevel categorical variables, SMDs are reported for each indicator level; for binary variables, the SMD is displayed for the non-reference level. An absolute SMD < 0.10 was considered indicative of acceptable covariate balance.

**Supplementary Table S3. Multivariable regression analyses in the sensitivity-matched cohort incorporating operating surgeon and year of surgery**

| **Variable** | **Blood loss (mL), β (95% CI)** | **P value** | **Operative time (h), β (95% CI)** | **P value** | **Surgeon-rated fatigue score, β (95% CI)** | **P value** | **Patient-reported scar satisfaction score, β (95% CI)** | **P value** |
| --- | --- | --- | --- | --- | --- | --- | --- | --- |
| Surgical approach (dual-port vs single-port) | -16.26 (-31.60, -0.92) | 0.038 | -0.09 (-0.23, 0.04) | 0.181 | -0.89 (-1.20, -0.59) | <0.001 | -1.59 (-3.68, 0.50) | 0.132 |
| Age | -0.61 (-1.69, 0.47) | 0.265 | 0.00 (-0.01, 0.01) | 0.412 | 0.00 (-0.02, 0.03) | 0.753 | -0.07 (-0.23, 0.10) | 0.416 |
| BMI | 1.68 (-0.82, 4.17) | 0.186 | 0.02 (-0.01, 0.04) | 0.183 | -0.06 (-0.12, -0.01) | 0.022 | -0.15 (-0.51, 0.21) | 0.404 |
| Fibroid diameter | 15.26 (9.71, 20.82) | <0.001 | 0.11 (0.06, 0.16) | <0.001 | 0.11 (0.00, 0.21) | 0.046 | -0.18 (-0.83, 0.46) | 0.571 |
| Posterior fibroid location | -6.74 (-22.81, 9.32) | 0.408 | 0.02 (-0.12, 0.17) | 0.731 | -0.11 (-0.44, 0.22) | 0.504 | -0.91 (-3.12, 1.30) | 0.413 |
| Pelvic adhesion grade | 9.40 (-2.88, 21.69) | 0.132 | -0.01 (-0.12, 0.10) | 0.882 | -0.19 (-0.43, 0.05) | 0.114 | 0.00 (-1.69, 1.69) | 0.998 |
| Medical comorbidity | -37.60 (-72.28, -2.92) | 0.034 | 0.25 (-0.06, 0.56) | 0.110 | 1.20 (0.37, 2.04) | 0.005 | 0.91 (-4.22, 6.03) | 0.724 |
| Previous pelvic surgery | -6.39 (-16.83, 4.05) | 0.228 | -0.01 (-0.10, 0.08) | 0.832 | -0.11 (-0.31, 0.09) | 0.296 | 0.03 (-1.31, 1.38) | 0.963 |
| Surgeon: Surgeon 2 vs Surgeon 1 | 52.51 (25.61, 79.40) | <0.001 | 0.15 (-0.09, 0.39) | 0.220 | 0.05 (-0.46, 0.56) | 0.842 | 0.76 (-4.04, 5.55) | 0.753 |
| Surgeon: Surgeon 3 vs Surgeon 1 | 40.68 (13.94, 67.42) | 0.003 | 0.35 (0.11, 0.59) | 0.005 | 0.40 (-0.12, 0.91) | 0.129 | 1.58 (-3.08, 6.23) | 0.500 |
| Year of surgery | -9.54 (-25.14, 6.07) | 0.229 | -0.05 (-0.19, 0.09) | 0.507 | -0.14 (-0.51, 0.22) | 0.435 | 0.98 (-1.11, 3.07) | 0.352 |

BMI, body mass index; CI, confidence interval. The single-port group, Surgeon 1, and the year 2024 were used as the reference categories for surgical approach, surgeon, and year of surgery, respectively. Regression models were adjusted for surgical approach, age, BMI, fibroid diameter, posterior fibroid location, pelvic adhesion grade, medical comorbidity, previous pelvic surgery, operating surgeon, and year of surgery.

**Supplementary sensitivity analyses and regression diagnostics**

Sensitivity analyses were performed in the propensity score-matched cohort using available cases for outcomes with incomplete assessment. The dual-port group was compared with the single-port group as the reference.

**Supplementary Table S4. Sensitivity analyses using alternative statistical approaches**

| **Outcome** | **Sensitivity model** | **Effect estimate (95% CI)** | **P value** | **N** | **Matched pairs/clusters** | **Conclusion** |
| --- | --- | --- | --- | --- | --- | --- |
| Blood loss | Log-transformed linear regression with pair-clustered CR2 standard errors | GMR = 0.756 (95% CI 0.614–0.930); relative change = -24.4% | 0.009 | 189 | 95 | Consistent with the primary analysis |
| Fatigue | Ordinal logistic regression with pair-clustered robust standard errors | OR = 0.052 (95% CI 0.012–0.227) | <0.001 | 110 | 79 | Consistent with the primary analysis |
| Scar satisfaction | Ordinal logistic regression with pair-clustered robust standard errors | OR = 0.336 (95% CI 0.129–0.876) | 0.026 | 78 | 64 | Consistent with the primary analysis |

GMR, geometric mean ratio; OR, odds ratio; CI, confidence interval. For blood loss, a GMR below 1 indicates lower blood loss in the dual-port group. For fatigue and scar satisfaction, higher scores were considered worse outcomes; therefore, OR below 1 indicates lower odds of being in a worse category in the dual-port group.

**Supplementary Table S5. Regression diagnostic summary for the primary linear models**

| **Model** | **N** | **Shapiro-Wilk P** | **Breusch-Pagan P** | **Heteroskedasticity** | **Maximum Cook's D** | **Influential observations** | **Maximum VIF/adjusted GVIF** | **Collinearity** |
| --- | --- | --- | --- | --- | --- | --- | --- | --- |
| Blood loss | 189 | <0.001 | 0.031 | Potential heteroskedasticity | 0.404 | 15 observation(s) > 4/n; none > 1 | 1.32 | Acceptable (<5) |
| Operative time | 190 | 0.116 | 0.084 | No statistical evidence | 0.084 | 13 observation(s) > 4/n; none > 1 | 1.32 | Acceptable (<5) |
| Fatigue | 110 | <0.001 | 0.034 | Potential heteroskedasticity | 0.247 | 9 observation(s) > 4/n; none > 1 | 1.24 | Acceptable (<5) |
| Scar satisfaction | 78 | 0.054 | 0.271 | No statistical evidence | 0.088 | 2 observation(s) > 4/n; none > 1 | 1.13 | Acceptable (<5) |

Residual normality should be judged primarily from the Q-Q plots rather than the Shapiro-Wilk test alone. A Breusch-Pagan P value below 0.05 suggests potential heteroskedasticity. Cook's distance above 4/n was used as a screening threshold, whereas values above 1 indicate greater concern. For multi-degree-of-freedom factors, GVIF^(1/(2*Df)) was used; for single-degree-of-freedom terms, conventional VIF was used.

**Supplementary Table S6. Characteristics according to availability of surgeon-rated fatigue score**

| **Characteristic** | **Fatigue score available (n = 110)** | **Fatigue score missing (n = 80)** | **P value** |
| --- | --- | --- | --- |
| **Surgical approach, n (%)** |  |  | 0.463 |
| Single-port | 58 (52.7%) | 37 (46.2%) |  |
| Dual-port | 52 (47.3%) | 43 (53.8%) |  |
| Age, years | 37.66 ± 7.13 | 38.23 ± 8.19 | 0.669 |
| BMI, kg/m² | 22.77 ± 3.38 | 22.70 ± 3.48 | 0.617 |
| Fibroid diameter, cm | 6.57 ± 1.42 | 6.68 ± 1.36 | 0.385 |
| **Fibroid location, n (%)** |  |  | 0.882 |
| Non-posterior wall | 65 (59.1%) | 46 (57.5%) |  |
| Posterior wall | 45 (40.9%) | 34 (42.5%) |  |
| Pelvic adhesion grade | 0.60 ± 0.86 | 0.51 ± 0.66 | 0.898 |
| **Medical comorbidity, n (%)** |  |  | 0.745 |
| No | 105 (95.5%) | 75 (93.8%) |  |
| Yes | 5 (4.5%) | 5 (6.2%) |  |
| Number of previous pelvic surgeries | 0.62 ± 0.82 | 0.61 ± 0.80 | 0.996 |
| **Operating surgeon, n (%)** |  |  | 0.833 |
| Surgeon 1 | 22 (20.0%) | 14 (17.5%) |  |
| Surgeon 2 | 43 (39.1%) | 30 (37.5%) |  |
| Surgeon 3 | 45 (40.9%) | 36 (45.0%) |  |
| **Year of surgery, n (%)** |  |  | **<0.001** |
| 2024 | 83 (75.5%) | 7 (8.8%) |  |
| 2025 | 27 (24.5%) | 73 (91.2%) |  |
| Estimated blood loss, mL | 66.15 ± 69.43 | 54.00 ± 37.71 | 0.940 |
| Operative time, h | 1.77 ± 0.54 | 1.68 ± 0.42 | 0.383 |
| Time to first oral intake, h | 2.19 ± 1.51 | 2.35 ± 0.98 | **0.029** |
| Time to first ambulation, h | 2.74 ± 0.88 | 3.11 ± 1.02 | **0.033** |
| Time to first voiding, h | 3.06 ± 0.97 | 3.33 ± 1.15 | 0.141 |
| Time to first flatus, h | 11.87 ± 4.87 | 11.27 ± 4.97 | 0.508 |
| Length of hospital stay, days | 1.05 ± 0.27 | 1.00 ± 0.00 | 0.055 |
| Postoperative pain score (VAS) | 1.77 ± 0.74 | 1.69 ± 0.85 | 0.486 |
| **Analgesic use, n (%)** |  |  | 1.000 |
| No | 109 (99.1%) | 79 (98.8%) |  |
| Yes | 1 (0.9%) | 1 (1.2%) |  |
| **Postoperative complication, n (%)** |  |  | 1.000 |
| No | 109 (99.1%) | 80 (100.0%) |  |
| Yes | 1 (0.9%) | 0 (0.0%) |  |

Data are presented as mean ± standard deviation or n (%). Continuous variables were compared using the Wilcoxon rank-sum (Mann–Whitney U) test. Binary categorical variables were compared using Fisher’s exact test, and multilevel categorical variables using the chi-square test; Monte Carlo simulation was used when expected cell counts were small. P values are nominal and two-sided.

**Supplementary Table S7. Characteristics according to availability of patient-reported scar satisfaction score**

| **Characteristic** | **Scar satisfaction score available (n = 78)** | **Scar satisfaction score missing (n = 112)** | **P value** |
| --- | --- | --- | --- |
| **Surgical approach, n (%)** |  |  | 0.461 |
| Single-port | 36 (46.2%) | 59 (52.7%) |  |
| Dual-port | 42 (53.8%) | 53 (47.3%) |  |
| Age, years | 37.05 ± 7.09 | 38.49 ± 7.88 | 0.193 |
| BMI, kg/m² | 22.48 ± 3.14 | 22.93 ± 3.59 | 0.418 |
| Fibroid diameter, cm | 6.74 ± 1.62 | 6.53 ± 1.21 | 0.830 |
| **Fibroid location, n (%)** |  |  | 0.882 |
| Non-posterior wall | 45 (57.7%) | 66 (58.9%) |  |
| Posterior wall | 33 (42.3%) | 46 (41.1%) |  |
| Pelvic adhesion grade | 0.62 ± 0.72 | 0.53 ± 0.82 | 0.189 |
| **Medical comorbidity, n (%)** |  |  | 0.530 |
| No | 75 (96.2%) | 105 (93.8%) |  |
| Yes | 3 (3.8%) | 7 (6.2%) |  |
| Previous pelvic surgeries | 0.60 ± 0.80 | 0.62 ± 0.83 | 0.909 |
| **Operating surgeon, n (%)** |  |  | 0.870 |
| Surgeon 1 | 14 (17.9%) | 22 (19.6%) |  |
| Surgeon 2 | 29 (37.2%) | 44 (39.3%) |  |
| Surgeon 3 | 35 (44.9%) | 46 (41.1%) |  |
| **Year of surgery, n (%)** |  |  | 0.770 |
| 2024 | 38 (48.7%) | 52 (46.4%) |  |
| 2025 | 40 (51.3%) | 60 (53.6%) |  |
| Estimated blood loss, mL | 63.85 ± 61.71 | 59.01 ± 56.03 | 0.667 |
| Operative time, h | 1.75 ± 0.52 | 1.72 ± 0.47 | 0.924 |
| Time to first oral intake, h | 2.23 ± 1.01 | 2.27 ± 1.51 | 0.577 |
| Time to first ambulation, h | 3.00 ± 1.01 | 2.81 ± 0.91 | 0.522 |
| Time to first voiding, h | 3.27 ± 1.17 | 3.09 ± 0.95 | 0.608 |
| Time to first flatus, h | 12.01 ± 5.26 | 11.39 ± 4.61 | 0.421 |
| Length of hospital stay, days | 1.03 ± 0.16 | 1.04 ± 0.23 | 0.958 |
| Postoperative pain score (VAS) | 1.70 ± 0.85 | 1.76 ± 0.75 | 0.522 |
| **Analgesic use, n (%)** |  |  | 0.513 |
| No | 78 (100.0%) | 110 (98.2%) |  |
| Yes | 0 (0.0%) | 2 (1.8%) |  |
| **Postoperative complication, n (%)** |  |  | 1.000 |
| No | 78 (100.0%) | 111 (99.1%) |  |
| Yes | 0 (0.0%) | 1 (0.9%) |  |

Data are presented as mean ± standard deviation or n (%). Continuous variables were compared using the Wilcoxon rank-sum (Mann–Whitney U) test. Binary categorical variables were compared using Fisher’s exact test, and multilevel categorical variables using the chi-square test; Monte Carlo simulation was used when expected cell counts were small. P values are nominal and two-sided.

**Supplementary Figures**

**Supplementary Figure S1. Covariate balance before and after propensity score matching.**
Absolute standardized mean differences (SMDs) for baseline covariates are shown before and after propensity score matching. Triangles represent pre-matching values, and circles represent post-matching values. The solid vertical line indicates an absolute SMD of 0, and the dashed vertical line indicates the balance threshold of 0.10. An absolute SMD below 0.10 was considered indicative of acceptable covariate balance.

**Supplementary Figure S2. Sensitivity analyses for blood loss, surgeon-rated fatigue, and patient-reported scar satisfaction.**

Point estimates represent the geometric mean ratio (GMR) for log-transformed blood loss and odds ratios (ORs) for the ordinal fatigue and scar-satisfaction outcomes. Horizontal lines indicate 95% confidence intervals. The dashed vertical line indicates the null value (GMR or OR = 1).

**Supplementary Figure S3. Propensity score distributions before and after primary matching.**

Density plots show the distribution of estimated propensity scores in the single-port and dual-port groups before and after 1:1 nearest-neighbor matching. Greater overlap after matching indicates improved propensity-score balance and common support between the treatment groups.
